# SACCADES TO SCREEN AND ASSESS COGNITIVE IMPAIRMENT IN OLDER ADULTS: A SYSTEMATIC REVIEW AND META-ANALYSIS

**DOI:** 10.1093/geroni/igac059.1868

**Published:** 2022-12-20

**Authors:** Qiaoqin Wan, Shifang Zhang, Xiuxiu Huang, Xiaoyan Zhao

**Affiliations:** Peking University, Beijing, Beijing, China (People's Republic); Peking University, Beijing, Beijing, China (People's Republic); Peking University, Beijing, Beijing, China (People's Republic); Peking University, Beijing, Beijing, China (People's Republic)

## Abstract

**Objective:**

To systematically summarize the evidence of saccade as a screening and assessing for patients with mild cognitive impairment (MCI) and dementia.

**Methods:**

English databases including PubMed, EMBASE, the Cochrane Library, Web of science, and PsycINFO and Chinese databases including CNKI, Wanfang and VIP were searched. Studies that analyzed the metrics of saccade in people with health cognition, MCI, or dementia were included. The quality of the included studies was evaluated with Cross-sectional/ Prevalence Study Quality from Agency for Healthcare Research and Quality (AHRQ). Study characteristics, participants' characteristics, sample size, saccade procedure, and metrics were extracted from the included studies.

**Results:**

Twenty-two studies involving 1595 participants were included. Meta-analysis showed that peak velocity (SMD= -0.27°/s, 95% CI (-0.44, -0.11), latency (SMD=-0.36ms , 95%CI (-0.51,-0.20), and accuracy rate (SMD=0.42%, 95%CI (0.17,0.68) of prosaccade between older adults with and without cognitive impairment had significant difference. The performance in latency (SMD=-0.56ms, 95%CI(-0.72,-0.39), accuracy rate (SMD=1.32%, 95%CI(1.07,1.56), and corrected errors (SMD=1.23%, 95%CI(0.98,1.47) of antisaccade in people with health cognition was better than that in older adults with cognitive impairment. The results of subgroup analysis revealed that the accuracy rate of prosaccade, latency and accuracy rate of antisaccade demonstrated crucial difference between health older adults and people with MCI, while only accuracy rate of antisaccade showed significant difference between people with MCI and dementia.

**Conclusions:**

The metrics of saccade, especially antisaccade, can be a potential screening and assessing tool for MCI and dementia in elderly persons.

